# Patent Medicines

**Published:** 1851-11

**Authors:** 


### PATENT MEDICINES.

The increase of the patent medicine business in this country is a crying evil that should be arrested, and we know of no means so effectual as the fixed determination on the part of the profession, to have nothing to do with anything that is connected with the base and dishonorable business. The use that is constantly made of the names of respectable physicians, in order to promote the sale of the abominations of the miserable charlatans and quacks of the day, should teach the medical profession a double lesson: 1st. That the quack feels dependent on med ical men for imposing his nostrums on the community: 2dly. That it is the duty of medical men to take such a stand in relation to nostrums, that when the community see the authority of the profession invoked in aid of quack medicines, and the names of physicians bandied about as friendly to the special nostrum be-praised, they should know that the statement must necessarily be false. The impudence of quacks is beyond all conception. For instance: the venders of Ayers' Cherry Pectoral have for years published what purports to be a certificate from Prof. Benjamin Silliman, senr., setting forth the wonderful merits of said Pectoral, and professing to give the personal experience of the Professor in the use of the nostrum in his own family. The imposition at length reached the impudence of publishing this certificate in the New Haven papers, and Prof. Silliman thought it due to himself, to put an extinguisher upon the deliberate forgery. He had never had a drop of the Pectoral in his family, never knew anything whatever of its qualities, and of course never gave a certificate to any one connected with the nostrum. The facts the Professor set forth before the public, and requested the managers of the Ayers' humbug to discontinue the use of his name. But Mr. Ayers' found too much benefit from the forgery, of Professor Silliman's authority, and he continues, to the present time, to publish what he calls a certificate from Professor Silliman.

Physicians are sometimes made acquainted with the formula, or what is represented to be the formula of some nostrum, and they may conclude that such a combination may be useful in some disordered conditions of the system. A specimen of the nostrum is sent to them, and forthwith their names are paraded in the advertisements of the quack, as prescribers of his nostrum, the physicians making a special use of the formula, and the quack using their names as authority for his statement, that his medicine is a universal panacea.

Every physician owes a sacred duty to his profession, and to humanity, and that is never, under any circumstances, to have anything to do with any quack nostrum. B.

